# Bilateral common carotid artery ultrasound for prediction of incident strokes using intima-media thickness and external diameter: an observational study

**DOI:** 10.1186/1476-7120-11-22

**Published:** 2013-06-15

**Authors:** Marsha L Eigenbrodt, Gregory W Evans, Kathryn M Rose, Zoran Bursac, Richard E Tracy, Jawahar L Mehta, David J Couper

**Affiliations:** 1 College of Medicine and Fay W. Boozman College of Public Health, University of Arkansas for Medical Sciences, Little Rock, AR, USA; 2 Division of Public Health Sciences, Wake Forest School of Medicine, Winston-Salem, NC, USA; 3 Department of Epidemiology, Gillings School of Global Public Health, University of North Carolina, Chapel Hill, NC, USA; 4 SRA International, Inc., Durham, NC, USA; 5 Department of Biostatistics, Fay W. Boozman College of Public Health, University of Arkansas for Medical Sciences, Little Rock, AR, USA; 6 Department of Pathology, Louisiana State University Health Science Center, New Orleans, LA, USA; 7 Departments of Medicine, Physiology, and Biophysics, University of Arkansas for Medical Sciences, Little Rock, AR, USA; 8 Department of Biostatistics, Gillings School of Global Public Health, University of North Carolina, Chapel Hill, NC, USA

**Keywords:** Stroke, Atherosclerosis, Carotid arteries, Ultrasound, Intima-media thickness, Arterial diameter, Arterial geometry, Bilateral, Prospective study

## Abstract

**Background:**

External common carotid artery (CCA) diameter and intima-media thickness (IMT) are independently associated with incident stroke and other cardiovascular events. Arterial geometry such as large IMT and large diameter may reflect vulnerable plaques and so impact stroke risk. Finally, arterial changes that exist bilaterally may increase stroke risk.

**Method:**

We studied middle-aged men and women (n=7276) from a prospective observational study who had right (R) and left (L) CCA IMT and external diameters measured via B-mode ultrasound (1987–89) in order to categorize CCA geometry. Using side- and gender-specific IMT and diameter medians, we categorized each measurement as large (≥ median) vs. not large (< median) and defined four geometries: both IMT and diameter were large, only one parameter was large, or neither was large (reference group). Participants were followed for first time stroke through December 31, 1999. We used proportional hazards models to assess associations between right and left CCA geometries with new stroke. We also calculated positive and negative likelihood ratios (+LR and -LR) for CCA bilateral phenotypes as a measure of diagnostic accuracy.

**Results:**

Presence of both large CCA IMT and large diameter on one side was associated with strong stroke risk even after risk factor adjustment (men: RCCA hazard ratio [HR]=3.7 95% confidence interval [CI]=1.9-7.4; LCCA HR=2.4 95% CI=1.4-4.4; women: RCCA HR=4.0 95% CI=1.5-10.5; LCCA HR=5.7 95% CI=1.7-19.0). Presence of both large IMT and large diameter bilaterally was the strongest predictor of stroke identifying 64% of women and 44% of men who developed strokes. This phenotype showed potential for predicting stroke among individuals (women: +LR=3.1, 95% CI=2.6-3.8; men: +LR=2.3, 95% CI=1.8-2.8).

**Conclusion:**

Bilateral carotid artery geometries may be useful for stroke risk prediction.

## Background

Stroke is a leading cause of death and adult disability in the US [1,2]. About 88% of strokes in Western countries are ischemic [3] with almost 50% of strokes occurring in the presence of atherosclerosis of the extracranial or large intracranial arteries [4]. Significant stenosis of large arteries accounts for only 20% of ischemic strokes overall [3] and only 10% in asymptomatic people [4]. Vulnerable plaques, which may rupture to produce strokes [4], are frequently found in arteries with less than 50% lumen stenosis [5] and have been associated with ispsilateral cryptogenic strokes [6]. So, markers for stroke other than stenosis are needed [7].

Models for predicting overall cardiovascular disease (CVD) risk [8-10] and stroke-specific risk [11,12] are generally acceptable, but do have limitations [8,13,14] such as limited risk factor measurements, and assuming risk factor effects are consistent across ages and in different risk factor groups. In particular, better stratification models for women and others with low short-term, but high lifetime risk are needed [13,15]. Direct vascular examination has been proposed as a way to improve stroke prediction in individuals [7,16]. Modest improvements in prediction were gained by adding various carotid IMT measures to CVD [17,18] and stroke [11,17] prediction models. Though not tested in the stroke prediction model, carotid stiffness measures derived from carotid diameters were independently associated with stroke [19]. A promising, but expensive method is high resolution MRI [7] which can identify carotid plaque characteristics that increase a person’s stroke risk [5,7]. Hypertension, the most powerful predictor of both lacunar and cortical infarcts [20] has been associated with increased elastic arterial diameters, as has metabolic syndrome and other risk factors [21,22]. Likewise, outward remodeling occurs more frequently in areas of vulnerable plaques [23]. So, arterial diameter enlargement could help identify stroke risk as suggested in a case control study where carotid diameters were larger in stroke than in non-stroke patients [24]. In fact, a recent prospective study found that both intima-media thickness (IMT) and external common carotid artery (CCA) diameter were independently associated with incident stroke and that diameter added discrimination [25]. However, external artery diameter may reflect different underlying mechanisms/characteristics depending upon the coexisting wall thickness [26,27]. Thus, we evaluated four CCA geometries on the right and left sides defined by B-mode ultrasound assessment of both IMT and external diameter, and then we explored bilateral combinations of these arterial patterns as phenotypes for risk discrimination.

## Subjects and methods

Sample: The Atherosclerosis Risk in Community (ARIC) Study is a prospective study of 15792 men and women who were 45–64 years of age at baseline (1987–89). Participants are predominantly black and white and were recruited from four U. S. communities [28]. All participants signed informed consents. Committees for human subjects’ protection at each participating site approved the original ARIC study, and the current ancillary study was approved at the University of Arkansas for Medical Sciences. NHLBI provided the ARIC limited access data (ARICLAD) for this study. The ARICLAD contains 99.6% (n=15732) of the individuals from the full ARIC study. Not all participants had usable right and left CCA measurements of IMT and external diameter (diameter), and because the missing LCCA and RCCA parameters overlapped only partially, complete CCA IMT and diameter measures were available for only 10096 participants. After removing persons with missing computer algorithm-defined stroke at baseline, 7874 persons remained. Additional restrictions for missing information on baseline prevalent coronary heart (n=138), and other major potential confounders [age, sex, race, diabetes mellitus, systolic and diastolic blood pressure, current drinking or smoking status, low and high density lipoproteins (LDL and HDL), fibrinogen, glucose, white blood count, triglyceride, and hypertension status (n=299)] left 7437 in the baseline sample for assessing risk factor associations with right and left arterial geometries. For analyses of new strokes, the 161 persons with stroke at baseline were removed, leaving 7276 individuals for incidence analyses.

### Outcome: stroke definitions

The baseline definition of stroke that we used was ARIC’s computer algorithm definition based on six symptoms (speech, vision, double vision, numbness, paralysis and dizziness) [29]. Incident ischemic stroke events were identified from information collected during annual telephone interviews and from an ongoing community surveillance program and were validated and classified as ischemic vs. hemorrhagic using hospital and autopsy information [30]. The current study evaluates definite or probable ischemic strokes that include both thrombotic and cardio-embolic events that lasted more than 24 hours and which were not secondary to trauma, neoplasms, infections, vasculitis, or hematologic abnormalities.

### Main exposures

The main exposures, unilateral CCA geometric patterns and bilateral phenotypes, were defined based on ultrasound measurements of CCA IMT and external diameter (interadventitial distance) at baseline. The ARIC procedure for CCA ultrasound measurement has been previously described [21,31-33]. Briefly, ultrasound scans of three carotid artery segments from the right and the left were recorded for later reading according to a standardized protocol. All images were read at a central reading center using frames with the largest lumen (indicating systole) that had optimal arterial wall boundaries [33]. The right and left CCA far wall IMT and external diameter measures used in this study were the means of multiple measurements attempted at 1 mm intervals from the 1 cm carotid segment just proximal to the carotid bifurcation using standard interfaces and optimal angle. We used gender-specific medians of the RCCA and LCCA IMTs and external diameters from our study sample (Table 1) to categorize each parameter as large (upper 50^th^ percentile) or not large (lower 50^th^ percentile) to achieve comparably sized groups for comparisons of IMT and diameter effects. Then, four geometric patterns were defined for each CCA (IMT and diameter both large, only IMT large, only diameter large, and neither large=reference). Cross classification of R and L CCA patterns produced 16 categories that could be reduced to ten non-overlapping categories/phenotypes which might have different implications. For instance, we hypothesized that large diameter and large IMT on the same side may indicate vulnerable plaques, which if present bilaterally would indicate a high risk group. Large IMT in the absence of large diameter may indicate stable plaques that narrow the lumen. Vascular narrowing on both sides could produce cerebral ischemia in the presence of hypotensive episodes. Bilaterally large diameter in the absence of large IMT could indicate the presence of known factors such as hypertension [21,22] that also cause stroke. Unilateral abnormalities could indicate only focal change, or less definitive abnormalities. After assessing bilateral phenotypes, we then explored various combinations of these phenotypes as stroke predictors.

**Table 1:** Gender-specific medians for the right and left common carotid artery intima-media thicknesses and external diameters*

**Gender**	**Vascular parameter**	**Median**
Men (n=3330)		mm
RCCA	IMT	0.663
	Diameter	8.092
LCCA	IMT	0.676
	Diameter	7.997
Women (n=4107)		
RCCA	IMT	0.609
	Diameter	7.303
LCCA	IMT	0.603
	Diameter	7.199

*Determined by B-mode ultrasound.

RCCA and LCCA = right and left common carotid artery respectively.

*IMT* intima-media thickness.

### Other variables

Baseline plaques were identified from any right or left carotid segment (common, internal, and bifurcation) [31,32]. The methods for defining smoking, drinking and disease status, as well as methods for laboratory measures and for anthropometric measures have been summarized previously [11,32]. Diabetes mellitus was defined as a fasting glucose level ≥ 126 mg/dl (7.0 mmol/L), a non-fasting level ≥ 200 mg/dl (11.1 mmol/L) (hexokinase method), self-reported diagnosis of diabetes, or use of medication for diabetes. Baseline, sitting, systolic and diastolic blood pressures were the means of the second and third blood pressure values obtained using a standard sphygmomanometer. Hypertension was defined as blood pressure ≥ 140/90 mm Hg or use of anti-hypertensive medication within 2 weeks of the clinic visit. Smoking status and drinking status were self-reported. We used the ARIC definition of coronary heart disease (CHD) which includes myocardial infarction from the baseline electrocardiogram, a self-reported history of a myocardial infarction or of specific types of heart vascular surgery. Body mass index (BMI, weight in kg/height in m^2^) was calculated from baseline measurements. Central laboratories measured fibrinogen, total cholesterol, high density lipoprotein cholesterol and triglycerides, while low density lipoprotein cholesterol was computed. Local laboratories measured white blood counts.

### Statistical methods

Analyses were performed using SAS 9.1 except for diagnostic test evaluations which were performed using online software [34]. Because of the potential for gender differences in external diameter associations [35,36], we evaluated men and women separately. The Kruskal Wallis and chi-square tests were used to assess overall significance of variations in baseline characteristics across the arterial geometries. Post hoc comparisons were made using the Student’s t test and chi-square tests. Gender- and side-specific Cox-proportional hazards models [37] were used to assess the relationship between the LCCA and RCCA geometric patterns with incident ischemic stroke. Basic models included indicator variables for CCA geometries and adjusted for age, race, and standing height and were followed by multivariable models that retained covariates based on significance (p< 0.05) and confounding (10% change in the point estimate for any of three indicator variables for CCA geometries). Finally, stroke incidence, sensitivity, specificity, and unadjusted relative risks were evaluated for the bilateral arterial phenotypes formed from cross-classification of the right and left CCA geometric patterns. To assess their utility as indicators of stroke risk, positive and negative likelihood ratios (+LR and –LR respectively) and 95% confidence intervals (CI) were calculated using online software [34] for several phenotypes in our study and from numbers calculated from published stroke incidence and sensitivities and specificities for the top quintile of the Framingham general CVD risk scores and cerebrovascular risk scores [12].

## Results

Median values for IMT and diameter were slightly larger for men than women (Table 1). The percentages of persons categorized as having specific geometries were very similar on the right and left (Table 2). However, for individuals, the right and left geometries were often discrepant with the Kappa statistics indicating only fair agreement in CCA classification on the right and left.

**Table 2:** Prevalence and concordance* of right and left common carotid artery geometries^**† **^**at baseline**

**Large parameter(s)**	**Number (%)**		**% Observed right-left**
	**RCCA**	**LCCA**	Agreement* (95% CI)
Men (N=3330)			
IMT and diameter	1068 (32.1)	1074 (32.3)	41.8 (39.2-44.3)
IMT only	599 (18.0)	607 (18.2)	23.3 (20.7-26.1)
Diameter only	598 (18.0)	599 (18.0)	23.7 (21.1-26.5)
Neither	1065 (32.0)	1050 (31.5)	46.3 (43.7-48.9)
Women (N=4107)			
IMT and diameter	1307 (31.82)	1391 (33.9)	43.3 (40.9-45.6)
IMT only	751 (18.29)	795 (19.4)	24.7 (22.3-27.4)
Diameter only	755 (18.4)	668 (16.3)	26.6 (24.2-29.2)
Neither	1294 (31.5)	1253 (30.5)	47.9 (45.6-50.2)
All (N=7437)			
IMT and diameter	2375 (31.9)	2465 (33.2)	42.6 (40.9-44.3)
IMT only	1350 (18.2)	1402 (18.9)	24.1 (22.6-26.0)
Diameter only	1353 (18.2)	1267 (17.0)	25.3 (23.5-27.2)
Neither	2359 (31.7)	2303 (31.0)	47.2 (45.5-48.9)

Women: Kappa=0.38, 95% CI 0.36-0.40; Men Kappa=0.35, 95% CI 0.33-0.38; All: Kappa=0.37, 95% CI= 0.35-0.38).

*% having the characteristic in both the RCCA and LCCA if it is present on either side.

^†^ RCCA and LCCA (right and left common carotid artery) geometries are based on ultrasound measurements of IMT and diameters at baseline where large is defined as ≥ the gender- and side-specific medians for IMT and external diameter in Table 1.

Table 3 provides the baseline characteristics for persons exhibiting the side-specific arterial geometries. Persons who exhibited both large IMT and large diameter on the right or left tended to have the most detrimental collection of characteristics: a larger percentage had carotid lesions (plaques), hypertension, and diabetes mellitus and higher levels of many other detrimental risk factors, such as systolic blood pressure, than were found among persons without both large IMT and diameter. Women with this pattern also had much higher CHD prevalence than women with other arterial geometries. For men, CHD prevalence was similar when both IMT and diameter was large as when only IMT was large. Risk factors were also elevated in men and women with isolated large IMT or isolated large diameter compared to the respective reference group. For men, stroke prevalence did not vary by CCA geometry, but for women, stroke prevalence was higher when the LCCA diameter was large compared to the LCCA reference.

**Table 3:** Gender-specific percentages and means of baseline characteristics for right and left common carotid artery geometries

	**Mean±SD or n (%) for**				**Mean±SD or n (%) for**			
	RCCA Geometries defined by large* component(s):				LCCA Geometries defined by large* component(s):			
	**IMT +**	**IMT**	**Diameter**	**Neither**	**IMT +**	**IMT**	**Diameter**	**Neither**
	**Diameter**	**Only**	**Only**		**Diameter**	**Only**	**Only**	
**Women**	**N=1307**	**N=751**	**N=755**	**N=1294**	**N=1391**	**N=795**	**N=668**	**N=1253**
Age, y	55.9±5.5^†^	53.9±5.5^†^	53.5±5.6^†^	51.4±5.2	55.9±5.6^†^	53.8±5.6^†^	53.3±5.5^†^	51.5±5.2
Race, black	507 (38.8)^†^	226 (30.1)^†^	224 (29.7)^†^	262 (20.2)	507 (36.4)^†^	197 (24.8)	246 (36.8)^†^	269 (21.5)
CHD	43 (3.3)^†^	13 (1.7)^§^	8 (1.1)	7 (0.5)	41 (2.9)^†^	11 (1.4)	11 (1.6)^§^	8 (0.6)
Carotid lesions	554 (46.9)^†^	227 (34.6)^†^	193 (27.8)^§^	279 (23.7)	548 (44.0)^†^	241 (33.8)^†^	178 (29.1)	286 (25.1)
Hypertension	634 (48.5)^†^	199 (26.5)^†^	252 (33.4)^†^	236 (18.2)	659 (47.4)^†^	207 (26.0)^†^	229 (34.3)^†^	226 (18.0)
Diabetes mellitus	202 (15.5)^†^	64 (8.5)^†^	50 (6.6)^§^	58 (4.5)	194 (13.9)^†^	67 (8.4)^†^	56 (8.4)^‡^	57 (4.5)
Current smoker	357 (27.3)	164 (21.8)	230 (30.5)^‡^	319 (24.7)	391 (28.1)^§^	194 (24.4)	191 (28.6)^§^	294 (23.5)
Current drink	596 (45.6)^†^	394 (52.5)^†^	381 (50.5)^†^	794 (61.4)	654 (47.0)^†^	436 (54.8)	334 (50.0)^†^	741 (59.1)
Systolic BP mm Hg	128±22^†^	116±16^†^	121±19^†^	112±16	128±22^†^	116±15^†^	121±19^†^	113±16
Diastolic BP mm Hg	73±12^†^	71±10^§^	72±11^†^	70±10	73±12^†^	71±10^§^	73±11^†^	70±10
Height, cm	163.0±5.9^‡^	162.0±6.0	163±6.0^§^	162.3±5.7	162.9±6.0^‡^	161.9±5.8	163.1±5.9^‡^	162.3±5.8
BMI, kg/m^2^	28.0±5.7^†^	26.5±5.0^†^	27.3±5.8^†^	25.5±4.7	27.8±5.6^†^	26.5±5.1^†^	27.5±5.9^†^	25.5±4.7
Glucose, IU	6.32±2.94^†^	5.76±1.92^‡^	5.69±1.65^§^	5.51±1.27	6.21±2.76^†^	5.80±1.90^†^	5.80±2.05^†^	5.49±1.28
HDL, IU	1.46±0.43^†^	1.55±0.45	1.50±0.47^‡^	1.57±0.45	1.45±0.44^†^	1.54±0.45	1.54±0.47	1.57±0.43
LDL, IU	3.65±1.05^†^	3.56±1.05^†^	3.41±1.06^‡^	3.27±1.03	3.65±1.06^†^	3.54±1.04^†^	3.39±1.10^§^	3.27±0.99
Fibrinogen, mg/dL	314±71^†^	298±56	308±64^†^	293±57	315±69^†^	299±61^§^	303±62^†^	293±57
WBC, 1000s/mm^3^	6.00±1.86	5.72±1.76	6.01±1.92	5.86±1.79	6.00±1.88^‡^	5.94±1.81	5.89±1.87	5.80±1.77
Triglyceride, IU	1.35±0.67^†^	1.28±0.65^†^	1.27±0.63^‡^	1.18±0.60	1.35±0.67^†^	1.29±0.64^†^	1.24±0.64^§^	1.18±0.59
Prevalent stroke	37 (2.8)	11 (1.5)	20 (2.6)	25 (1.9)	32 (2.3)	10 (1.3)	28 (4.2)^‡^	23 (1.8)
**MEN**	**N=1068**	**N=599**	**N=598**	**N=1065**	**N=1074**	**N=607**	**N=599**	**N=1050**
Age, y	56.5±5.5^†^	55.1±5.6^†^	53.7±5.8^†^	52.4±5.4	56.4±5.5^†^	54.6±5.6^†^	53.9±5.7^†^	52.6±5.5
Race, black	311 (29.1)^†^	171 (28.5)^†^	131 (21.9)	218 (20.5)	294 (27.4)^‡^	147 (24.2)	160 (26.7)^§^	230 (21.9)
CHD	100 (9.4)^†^	59 (9.8)^†^	38 (6.4)	56 (5.3)	111 (10.3)^†^	58 (9.6)^†^	36 (6.0)	48 (4.6)
Carotid lesions	551 (58.7)^†^	264 (50.8)^†^	231 (43.3)^†^	324 (34.1)	546 (57.9)^†^	275 (52.0)^†^	216 (40.5)	333 (35.5)
Hypertension	466 (43.6)^†^	174 (29.0)^‡^	214 (35.8)^†^	235 (22.1)	470 (43.8)^†^	184 (30.3)^†^	208 (34.7)^†^	227 (21.6)
Diabetes mellitus	153 (14.3)^†^	76 (12.7)^†^	56 (9.4)^§^	67 (6.3)	152 (14.2)^†^	75 (12.4)^†^	61 (10.2)^‡^	64 (6.1)
Current smoker	344 (32.2)^†^	156 (26.0)	207 (34.6)^†^	263 (24.7)	361 (33.6)^†^	157 (25.9)	187 (31.2)^§^	265 (25.2)
Current drink	667 (62.5)	389 (64.9)	408 (68.2)	705 (66.2)	672 (62.6)^§^	395 (65.1)	398 (66.4)	704 (67.0)
Systolic BP, mm Hg	129±20^†^	119±16^‡^	123±19^†^	116±14	128±21^†^	120±16^†^	124±19^†^	116±14
Diastolic BP, mmHg	77±13^†^	74±11	76±12^§^	74±10	76±13^†^	75±11	76±12^†^	74±10
Height, cm	176.6±6.4^§^	175.7±6.5	177.5±6.4^†^	176.0±6.3	176.6±6.4^‡^	175.5±6.3	177.7±6.4^†^	175.8±6.4
BMI, kg/m^2^	27.6±4.1^†^	26.7±3.8^§^	26.9±3.9^‡^	26.3±3.5	27.5±4.0^†^	26.8±3.7^§^	27.0±3.9^†^	26.4±3.6
Glucose, IU	6.28±2.39^†^	6.21±2.34^†^	5.92±1.66^§^	5.74±1.09	6.25±2.34^†^	6.16±2.20^†^	5.98±1.77^§^	5.75±1.23
HDL, IU	1.17±0.37	1.16±0.34	1.21±0.43^§^	1.16±0.34	1.17±0.37	1.13±0.32^‡^	1.22±0.45^§^	1.17±0.34
LDL, IU	3.65±0.95^§^	3.71±0.97^†^	3.48±0.96	3.54±0.94	3.65±0.98^‡^	3.70±0.95^†^	3.52±0.96	3.53±0.92
Fibrinogen, mg/dL	305±67^†^	294±62^§^	295±61^§^	287±62	303±66^†^	294±63^§^	298±64^‡^	287±61
WBC, 1000s/mm^3^	6.38±1.98^†^	6.03±1.82	6.34±2.07^§^	6.07±1.83	6.47±2.06^†^	6.19±1.98	6.07±1.83	6.04±1.79
Triglyceride, IU	1.49±0.75^§^	1.45±0.74	1.47±0.78	1.40±0.72	1.51±0.78^†^	1.49±0.76^‡^	1.43±0.72	1.37±0.72
Prevalent stroke	26 (2.4)	13 (2.2)	12 (2.0)	17 (1.6))	21 (2.0)	15 (2.5)	13 (2.2)	19 (1.8)

N= the maximum number possible; some variables have lower numbers available. Fewer persons had carotid plaque information.

*Large is defined as ≥ the gender- and side-specific medians for IMT and external diameter in Table 1.

Where omnibus tests are significant, post hoc comparisons are made to the neither large IMT nor large diameter group using the Student’s t test and Chi Square test. P-values ^†^ <0.0005, ^‡^ <0.005, ^§^ 0.05.

**Figure 1:**
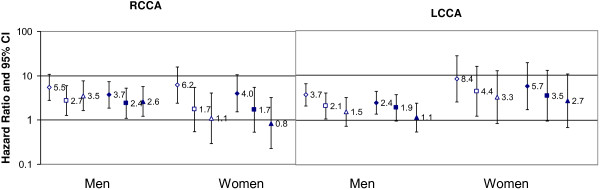
**Gender-specific hazard ratios and 95% confidence intervals (CI) for first incident ischemic stroke according to baseline common carotid artery (CCA) geometries of the right (RCCA) and left (LCCA) relative to persons with neither large IMT nor large external diameter on the respective side, ARICLAD 1987–1999.** Legend: Baseline gender- and side-specific median values for CCA IMT and external diameter were used to categorize each measure as large (≥ median) or not large (< median). For each side, diamonds indicate both IMT and diameter are large; squares indicate only IMT is large; triangles indicate only external diameter is large, and the reference group is neither IMT nor diameter is large. Open symbols indicate height, age, and race adjustment, while solid symbols indicate risk factor adjusted risks (covariates for men: baseline age, glucose, diastolic blood pressure, prevalent CHD, hypertension and current smoking; covariates for women RCCA: age, race, prevalent CHD, carotid plaques, triglycerides and its squared term, peripheral white blood count, and systolic blood pressure; LCCA model added body mass index and diabetes mellitus).

Between baseline (1987–1989) and the end of December 1999, 181 new strokes were documented, 66 among women and 115 among men (1.6% and 3.5% respectively). The stroke risks associated with separate RCCA and LCCA geometries are shown in Figure 1 Adjusting for age, race, and height, men and women having both large IMT and large diameter in one CCA at baseline had a strong risk of developing a stroke relative to the respective reference group and the risk remained large and statistically significant after risk factor adjustment. Also, while not statistically different, the hazard ratios were consistently larger than what was found for persons having isolated large IMT or diameter on the respective side.

Cross-classification of RCCA and LCCA geometries allowed evaluation of bilateral geometric CCA phenotypes (Table 4). Twenty percent of men and 21% of women had both large IMT and large diameter in the right and left CCA while almost 19% of men and women had neither large IMT nor large diameter on either side (reference). Considering the non-overlapping phenotypes in Table 4, the highest stroke cumulative incidence (7.7% for men and 4.9% for women) occurred in those with both IMT and diameter large bilaterally and low incidence was found in the reference groups where neither IMT nor diameter was large producing very large relative risks (women RR=16.3; men RR=6.4). In the absence of at least one bilaterally large parameter, stroke incidence was generally low.

**Table 4:** Incident strokes* for men and women with baseline bilateral common carotid artery phenotypes

**CCA Phenotypes:**	**Number**		**Incident stroke**	
**Ten non-overlapping phenotypes**	**Women**	**Men**	**Women N (%)**	**Men N (%)**
1) IMT and diameter both large bilaterally	*852*	*662*	*42 (4.9)*	*51 (7.7)*
2) IMT and diameter large on one side and opposite IMT large (bilaterally large IMT)	*271*	*229*	*4 (1.5)*	*12 (5.2)*
3) IMT and diameter large on one side and opposite diameter large (bilaterally large diameter)	*448*	*370*	*6 (1.3)*	*19 (5.1)*
4) IMT and diameter large on one side and neither opposite IMT nor diameter large	*206*	*172*	*1 (0.5)*	*1 (0.6)*
5) Bilaterally large IMT, *no large diameter*	*322*	*223*	*6 (1.9)*	*10 (4.5)*
6) Unilaterally large IMT *(R or L) and opposite diameter large*	*106*	*86*	*1 (0.9)*	*0*
7) Unilaterally large IMT *(R or L)* and no large diameter	*504*	*417*	*1 (0.2)*	*7 (1.7)*
8) Bilaterally large diameter, *no large IMT*	*269*	*223*	*1 (0.4)*	*5 (2.2)*
9) Unilaterally large diameter, (*R or L*)*, No large IMT*	*283*	*270*	*2 (0.7)*	*3 (1.1)*
10) No IMT or diameter large	*753*	*610*	*2 (0.3)*	*7 (1.2)*
All	4014	3262	66 (1.6)	115 (3.5)
**Combined phenotypes**				
IMT large bilaterally, regardless of diameter (Phenotypes:1,2,5)	1445	1114	52 (3.6)	73 (6.6)
Diameter large bilaterally, regardless of IMT (Phenotypes:1,3,8)	1569	1255	49 (3.1)	75 (6.0)
Large IMT bilaterally and/or large diameter bilaterally (Phenotypes: 1,2,3,5,8)	2162	1707	59 (2.7)	97 (5.7)
IMT and diameter both large on same side, unilaterally or bilaterally (Phenotypes: 1,2,3,4)	1777	1433	53 (3.0)	83 (5.8)
Large IMT, unilaterally or bilaterally, regardless of diameter (Phenotypes: 1-7)	2709	2159	61 (2.3)	100 (4.6)
Large diameter, unilaterally or bilaterally, regardless of IMT (Phenotypes: 1,2,3,4,6,8,9)	2435	2012	56 (2.5)	91 (4.7)

*First time strokes occurring after baseline (1987–89) through December 31, 1999 with follow up of 10 to 12 years.

Large is defined as ≥ the gender- and side-specific ultrasound medians for IMT and external diameter in Table 1.

*CCA* common carotid artery, *IMT* intima-media thickness.

 Table 5 presents diagnostic test information for selected bilateral arterial phenotypes. For the phenotype where CCA IMT and diameter were both large bilaterally, the sensitivity was 64% for women and 44% for men while specificity was about 80% for men and women. This resulted in +LRs of over 2 for men and approximately 3 for women. Expanding the positive test to include persons with any component large bilaterally (large IMT bilaterally and/or large diameter bilaterally) resulted in sensitivities greater than 80% for both men and women with improved -LRs that were comparable to those calculated for either Framingham scores. However, the reduced specificity produced +LRs that were not as good. The combined phenotype with the best sensitivity was any large IMT (large IMT unilateral or bilateral), but the low specificity resulted in lower +LR than the phenotype of large IMT and diameter bilaterally.

**Table 5:** Measures of stroke* risk assessment for selected bilateral CCA phenotypes in this study and for reported Framingham scores^**†**^

**Risk assessment criteria/phenotypes**	**Gender (#strokes)**	**Sensitivity**	**Specificity**	**Positive likelihood ratio (95% CI)**	**Negative likelihood ratio (95% CI)**
IMT and diameter both large, bilaterally	Men (51)	0.44	0.81	2.28 (1.84-2.84)	0.69 (0.59-0.81)
	Women (42)	0.64	0.79	3.10 (2.56-3.76)	0.46 (0.33-0.63)
IMT large bilaterally, regardless of diameter	Men (73)	0.63	0.67	1.92 (1.66-2.22)	0.55 (0.43-0.69)
	Women (52)	0.79	0.65	2.23(1.96-2.55)	0.33 (0.21-0.52)
Diameter large bilaterally, regardless of IMT	Men (75)	0.65	0.63	1.74 (1.51-2.00)	0.56 (0.43-0.71)
	Women (49)	0.74	0.61	1.93 (1.66-2.23)	0.42 (0.28-0.63)
IMT and diameter both large on same side, unilaterally or bilaterally	Men (83)	0.72	0.57	1.68 (1.49-1.90)	0.49 (0.36-0.65)
	Women (53)	0.80	0.56	1.84 (1.62-2.08)	0.35 (0.21-0.57)
Large IMT, unilaterally or bilaterally, regardless of diameter	Men (100)	0.87	0.35	1.33 (1.23-1.43)	0.38 (0.24-0.61)
	Women (61)	0.92	0.33	1.38 (1.28-1.48)	0.23 (0.10-0.53)
Large diameter, unilaterally or bilaterally, regardless of IMT	Men (91)	0.79	0.39	1.30 (1.18-1.43)	0.54 (0.37-0.77)
	Women (56)	0.85	0.40	1.41 (1.27-1.56)	0.38 (0.22-0.68)
Large IMT bilaterally and/or large diameter bilaterally	Men (97)	0.84	0.49	1.65 (1.51-1.80)	0.32 (0.21-0.49)
	Women (59)	0.89	0.47	1.68 (1.54-1.83)	0.23 (0.11-0.46)
**Framingham score: CVD**^†^	Men	0.72	0.81	3.85 (3.34-4.44)	0.34 (0.25-0.48)
	Women	0.62	0.81	3.23 (2.70-3.86)	0.47 (0.36-0.62)
**Cerebrovascular score**^†^	Men	0.76	0.81	4.1 (3.60-4.68)	0.29 (0.20-0.42)
	Women	0.64	0.81	3.36 (2.83-3.99)	0.44 (0.33-0.59)

*First time strokes occurring after baseline (1987–89) through December 31, 1999 with follow up of 10–12 years. ^†^The likelihood ratios for the Framingham scores are based on numbers calculated from the sensitivity and specificity reported for the top quintile of the Framingham scores in the Framingham population (12).

## Discussion

Most strokes, like other CVD, are caused by multiple factors that lead to atherosclerosis and/or small artery disease [2]. Current CVD risk stratification tools for general populations have limited success in persons with low short-term risk, but high lifetime risk [14] which comprises over 50% of US adults [15]. IMT and external diameter reflect the impact of multiple vascular risk factors and their interactions, treatment effects, the length of each exposure, and presence of carotid plaques. While our study has several limitations, the results indicate that bilateral measures of IMT and diameter are complementary and may help address stroke risk assessment, especially in women.

B-mode ultrasound is safe, easily administered and relatively low in cost [4]; thus, fulfilling several criteria for tools for screening asymptomatic persons [7,17]. We used mean IMT from the CCA far wall which is likely to be more clinically feasible than a mean including additional measurements from the bifurcation and internal carotid segments where more values were missing. Prior studies have shown measurements of mean far CCA wall IMT [33] and external diameter [38] to have acceptable repeatability. A reliability coefficient of 0.98 was found for the CCA mean arterial diameter and 0.78 for CCA maximum far wall thickness [38]. Side differences in measurement error were small [33]. Overall reliability coefficients (proportion of the between person variance over total variance) for the mean of the right and left CCA far wall IMT were calculated as 0.53 to 0.54 in a small subset with little between person variability, but was estimated to be 0.70 when the full ARIC between persons variability was considered [33]. Measurement errors usually bias results toward the null [33] and so should not have produced the large effects for the arterial geometries on the right and left. However, the discordance we found for the 50^th^ percentile categories on the right and left could have a component of measurement error. Consideration of both the right and left could thus improve prediction by assuring the true presence of larger parameters. However, as pointed out by Howard and co-authors who found discordance for continuous measures of right-left carotid IMT and diameter [39], much of the discordance is likely the result of the focal nature of the systemic atherosclerotic process.

That external carotid artery diameter added to stroke risk discrimination was shown previously by using diameter as a continuous measure [25]. Extending their study we found the strongest stroke risk was associated with the combined presence of large CCA IMT and large diameter. This change bilaterally was the phenotype with the highest cumulative stroke incidence. The +LR and -LR found for women with this phenotype, while not optimal, were comparable to those calculated for the reported sensitivities and specificities for the top quintiles of the Framingham scores [12]. No other phenotype had comparable specificity or +LR.

CHD and vulnerable plaques are important contributors to stroke [4,6]. We previously showed that the RCCA geometry of large IMT and large diameter was significantly associated with carotid plaques in any carotid segment [40] and with incident cardiac events [41]. Others have documented larger carotid diameters in the presence of some common types of vulnerable plaques [23] which could contribute to large IMT and large diameter’s association with incident strokes. Bilateral findings could also be important because it indicates more extensive disease and extent of intra- and extra-cranial atherosclerosis was recently shown to be associated with stroke occurrence in patients undergoing coronary artery bypass grafting [42]. The substantial stroke risk seen for bilaterally large IMT in the absence of large diameter may reflect the susceptibility to hypotension that can occur with narrowing of both carotids even in the absence of vulnerable plaques. However, given the associations were stronger in women and since during the period of study, ARIC was a relatively low risk population, one must consider alternative explanations.

Polak and co-authors found that CCA interadventitial distance was independently associated with left ventricular mass and proposed this association as an explanation for the ability of external diameter to improve prediction for cardiovascular events [36]. So, an alternative explanation for bilateral arterial phenotypes’ usefulness in stroke prediction may be the CCA patterns’ associations with major CVD risk factors. Persons with RCCA or LCCA that had combined large IMT and large diameter had less favorable risk factor distributions than other arterial geometries. Hypertension and increasing systolic blood pressure are associated with larger arterial diameters [21,22,36] and may contribute to strokes. External diameter is also larger with increasing age, smoking and presence of CHD [21,22], which are all major stroke risk factors [2,20]. The presence of atherosclerotic disease and risk factors increase the associated CCA external diameter size with age [35]. So, the presence of bilateral diameter enlargement may indicate major risk factors for lacunar and non-lacunar stroke. While height, age, and male gender are associated with larger arterial diameters [36], we controlled for those factors (and other risk factors) in the side-specific analyses and still found significant stroke associations for the large IMT plus large diameter geometry. This indicates carotid geometry provides information for stroke risk beyond multiple risk factors.

Our study indicates that CCA IMT and external diameter measures on the right and left are complementary in identifying stroke risk. The specificity for incident stroke was higher for the phenotype of both large IMT and large diameter on both sides at baseline than that for the other phenotypes resulting in a larger probability of true positive tests to probability of false positive tests. Of the bilateral phenotypes, the low -LR for large IMT bilaterally and or large diameter bilaterally, indicates a lower probability of false negative tests to the probability of true negative tests and could help identify low risk.

Limitations of the study need to be considered. We did not optimize the cuts for IMT and diameter in our population, but used medians as a simple means for defining which parameters were large. So, our model prediction is unlikely to be overly optimistic, but might be improved with optimal cuts. The specific sets of bilateral phenotypes were not preplanned. A major limitation is that the risk stratification was not validated internally or externally nor was there any formal statistical comparison to other risk stratification method. Further, there was no assessment of whether the geometric patterns or bilateral phenotypes could be used for reclassification of risk defined by the Framingham or other stratification methods. We also did not determine whether arterial geometry was associated with stroke independently of carotid stiffness measures.

Many ARIC participants were missing information on baseline algorithm defined stroke due to a change in the questionnaire [29]. Given that participants were randomly assigned an examination date, this should not have biased our sample. We were unable to adjust for geographic location. While the follow up for stroke in our study varied from about 10 to 12 years, the variation is unlikely to have biased the sensitivity, specificity or likelihood tests because initial evaluations, where the timing varied, were selected randomly.

Atrial fibrillation is often undiagnosed in the general population [2]. So, the prediction of strokes by CCA phenotypes in our study despite not adjusting for atrial fibrillation shows the potential strength of this risk stratification method. This strength may be explained by overlap of atrial fibrillation risk factors [43] with those for combined large IMT and large diameter. We were unable to test for confounding by atrial fibrillation in our study.

## Conclusion

Despite limitations, this study provides evidence that bilateral CCA patterns may be useful in identifying groups at high and low risk of stroke. However, our findings need to be confirmed in other populations and expanded to assess risk stratification improvement.

## Competing interests

The authors have no conflict of interest to report.

## Authors’ contributions

MLE (the corresponding author) conceived and designed the study (with input), performed many statistical analyses (with input), drafted the manuscript and provided multiple revisions. GWE provided input into the design of the study and into statistical analyses, provided input into revisions and knowledge of ARIC ultrasound studies. KMR provided input into multiple revisions and epidemiologic concepts, ARIC procedures, and multiple revisions. ZB performed some statistical analyses, provided input into statistical design and revisions of the manuscript. RET provided input into multiple revisions and important pathologic concepts potentially impacting associations. JLM provided cardiovascular disease knowledge and input into multiple revisions and into the final manuscript. DJC provided insight into statistical issues, input into multiple revisions and expertise on the ARIC data. All authors have read and approved submission of this manuscript.
